# Supplementary material for: Effect of Epichloë gansuensis Endophyte on Seed-Borne Microbes and Seed Metabolites in Achnatherum inebrians
Source: Microbiol Spectr. 2023 Feb 14;11(2):e01350-22. doi: 10.1128/spectrum.01350-22 (PMC10100691; doi:10.1128/spectrum.01350-22)
Supplement: Supplemental file 1 — Supplemental material. Download spectrum.01350-22-s0001.pdf, PDF file, 1.2 MB [file spectrum.01350-22-s0001.pdf]

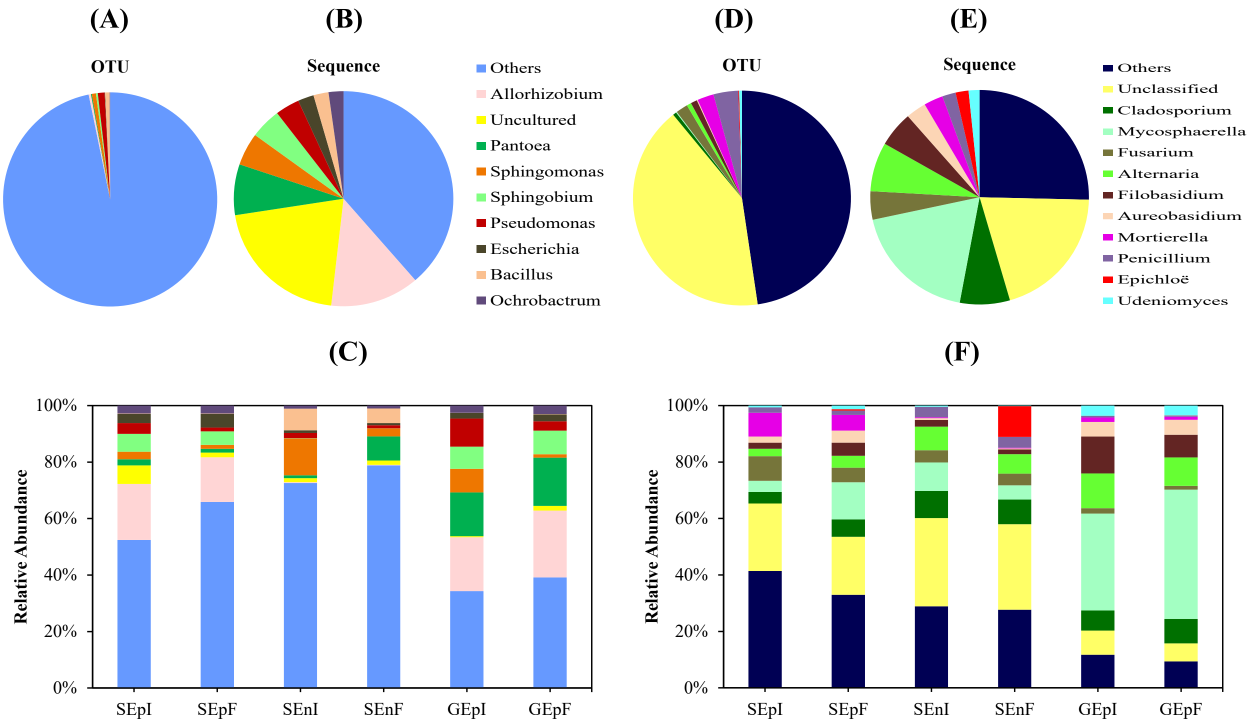

Figure S1. Taxonomic composition of microbial communities of seeds and glumes of *A. inebrians*

Operational taxonomic unit (OTU) of (A) bacteria and (D) fungi, sequence of (B) bacteria and (E) fungi in all samples and relative abundance of (C) bacteria and (F) fungi at the genus level located on or in *A. inebrians* seed and in endophyte treatments (SEpI (seed epiphytic microbes of the seed-borne of endophyte-infected plants), SEpF (seed epiphytic microbes of the seed-borne of endophyte-free plants), SEnI (seed endophytic microbes of the seed-borne of endophyte-infected plants), SEnF (seed endophytic microbes of the seed-borne of endophyte-free plants), GEpI (glume microbial community of endophyte-infected plants), GEpF (glume microbial community of endophyte-free plants), n=8). See also Table S1.

(A) (B)

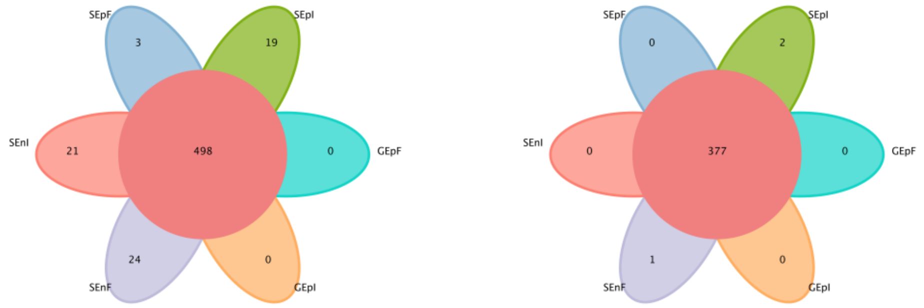

Figure S2. Venn diagram within seed and glume bacterial (A) and fungal (B) OTUs in and on EI and EF seeds and glumes of *Achnatherum inebrians*, (n=8).

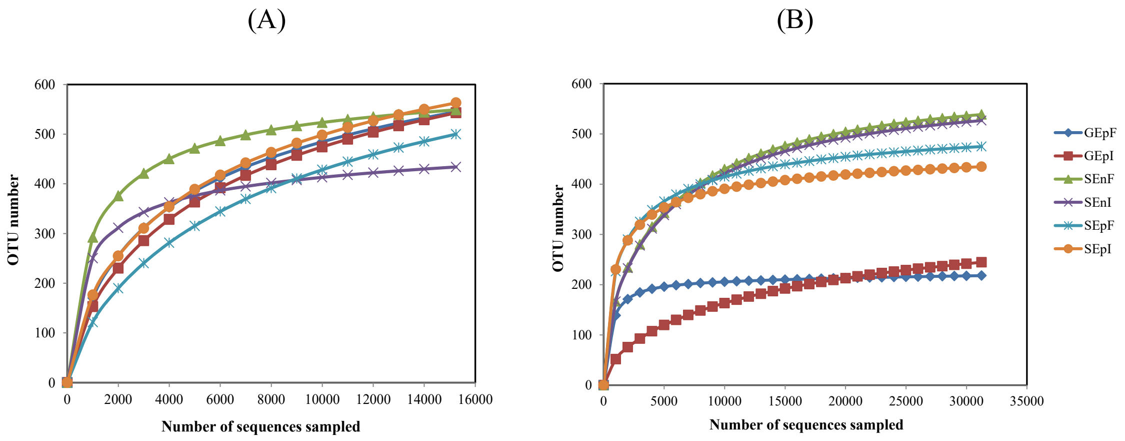

Figure S3. Rarefaction curves of the 16S rRNA and ITS gene sequence from the seed and glume bacteria (A) and fungi (B) in and on seeds and glumes of EI and EF *Achnatherum inebrians*, (n=8).

(A) (B)

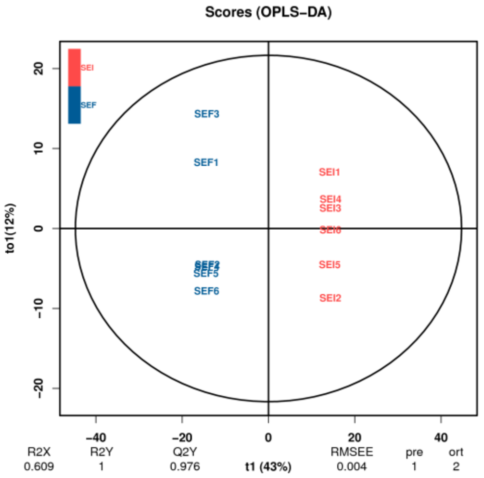

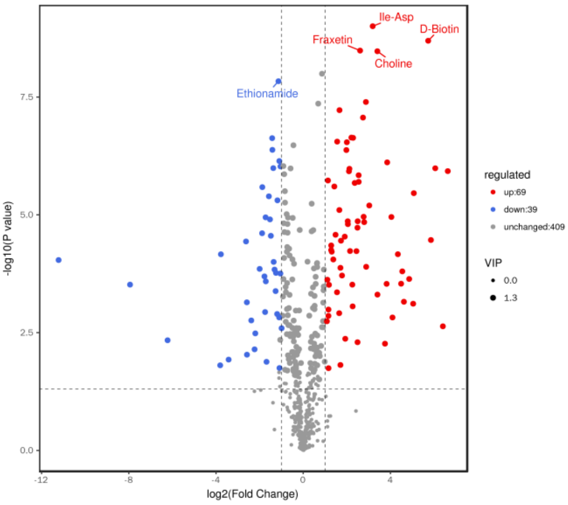

(C)

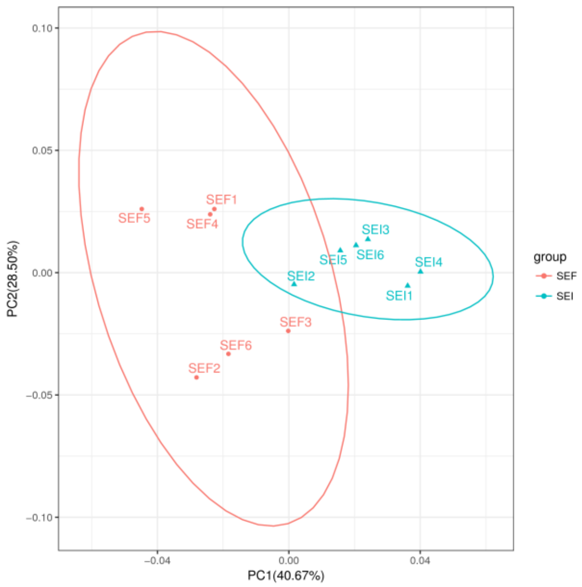

Figure S4. Identification of metabolites differentially accumulated in EI and EF of *Achnatherum inebrians* seeds. (A) The orthogonal projections to latent structures-discriminant analysis (OPLS-DA) score plot. (B) Volcano plot showing the increased and decreased metabolites between EI and EF. (C) Principal component analysis score plot based on the 517 detected metabolites, (n=6).

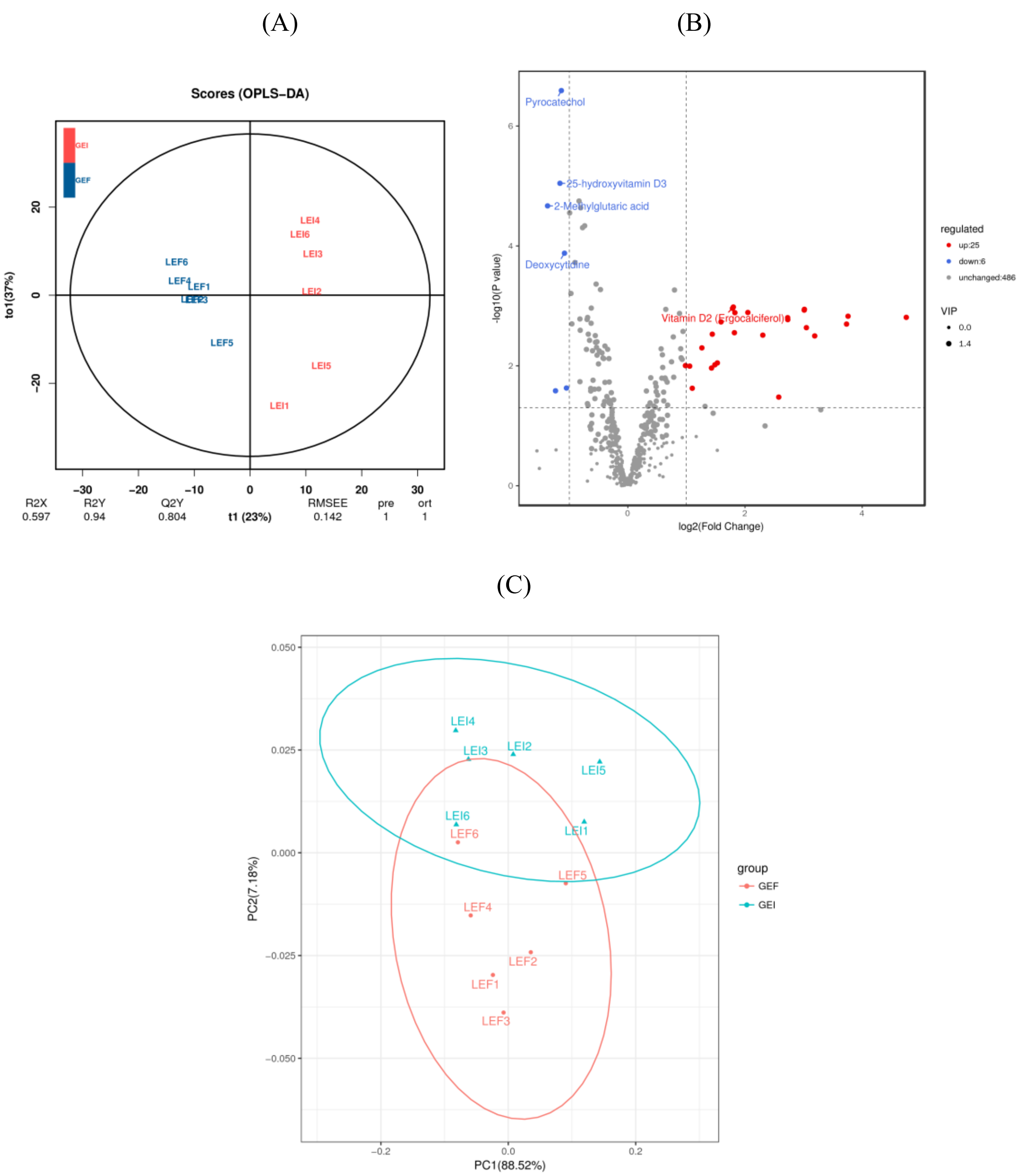

Figure S5. Identification of metabolites differentially accumulated in EI and EF glumes of *Achnatherum inebrians*. (A) The orthogonal projections to latent structures-discriminant analysis (OPLS-DA) score plot. (B) Volcano plot showing the increased and decreased metabolites between EI and EF. (C) Principal component analysis score plot based on the 517 detected metabolites, (n=6).

(A)

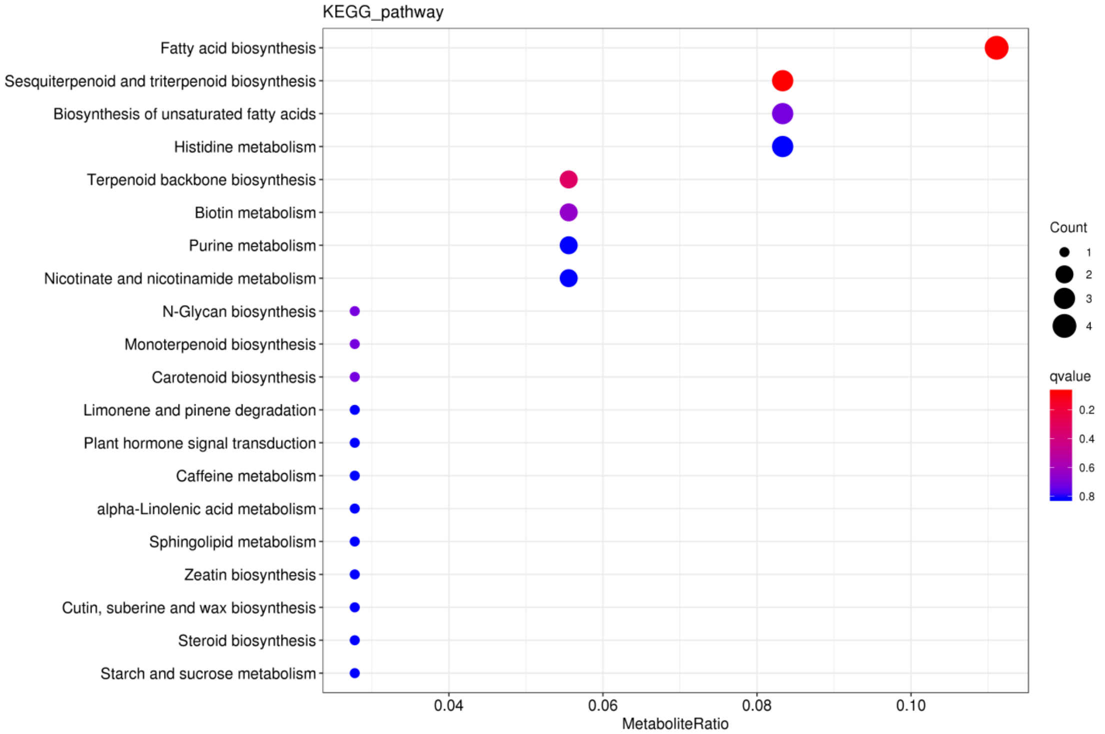

(B)

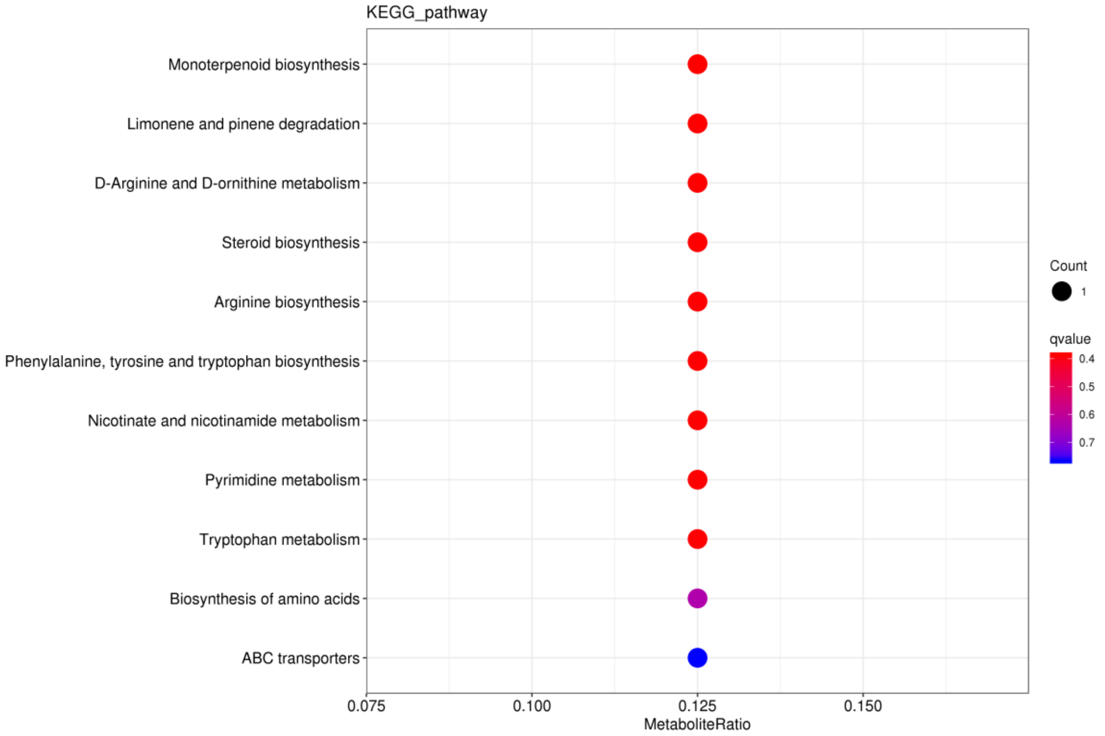

Figure S6. KEGG pathway enriched dotplot of differential metabolites in the EI as compared to the EF of seeds (A) and glumes (B) of *Achnatherum inebrians*.

Table S1. Seed and glume bacterial and fungal community composition in and on seeds and glumes of endophyte-infected (EI) and endophyte-free (EF) *Achnatherum inebrians* at the phylum level (SEpI (seed epiphytic microbes of the seed-borne of endophyte-infected plants), SEpF (seed epiphytic microbes of the seed-borne of endophyte-free plants), SEnI (seed endophytic microbes of the seed-borne of endophyte-infected plants), SEnF (seed endophytic microbes of the seed-borne of endophyte-free plants), GEpI (glume microbial community of endophyte-infected plants), GEpF (glume microbial community of endophyte-free plants), n=8).

Relative abundance (%)																		
Microbes	Phylum	SEpI	SEpF	SEnI	SEnF	GEpI	GEpF	OTU	Sequence	Genus	SEpI	SEpF	SEnI	SEnF	GEpI	GEpF	OTU	Sequence
Bacteria	Proteobacteria	58.4%	53.5%	45.6%	46.4%	75.8%	68.6%	493	424942	Others	52.4%	65.9%	72.6%	78.8%	34.4%	39.2%	1413	283130
	Actinobacteria	7.3%	18.0%	19.5%	19.9%	11.1%	8.8%	251	103269	*Allorhizobium*	19.8%	15.8%	0.3%	0.2%	19.0%	23.6%	5	96129
	Firmicutes	12.4%	9.6%	20.6%	15.5%	3.5%	5.9%	243	82368	Unclassified	6.6%	1.6%	1.4%	1.5%	0.4%	1.7%	0	151827
	Bacteroidetes	11.9%	3.8%	5.7%	6.5%	1.9%	4.4%	204	41724	*Pantoea*	2.2%	1.4%	1.0%	8.5%	15.6%	17.1%	2	55886
	Cyanobacteria	4.8%	5.6%	0.0%	0.0%	5.1%	9.6%	9	30647	*Sphingomonas*	2.7%	1.4%	13.0%	2.8%	8.3%	1.2%	9	35913
	Acidobacteria	1.3%	4.1%	1.8%	3.0%	1.1%	1.3%	89	15432	*Sphingobium*	6.3%	4.7%	0.1%	0.0%	7.9%	8.3%	4	33412
	Gemmatimonadetes	0.7%	1.1%	2.6%	4.0%	0.2%	0.1%	62	10595	*Pseudomonas*	3.8%	1.4%	1.9%	1.1%	10.0%	3.4%	15	26224
	Chloroflexi	0.5%	1.4%	1.6%	0.8%	0.3%	0.5%	34	6245	*Escherichia*	3.2%	4.9%	0.9%	0.7%	2.0%	2.4%	1	17216
	Nitrospirae	0.1%	0.2%	0.6%	1.4%	0.2%	0.0%	7	3106	*Bacillus*	0.2%	0.1%	7.7%	5.2%	0.1%	0.1%	10	16261
	Rokubacteria	0.1%	0.6%	0.5%	0.9%	0.2%	0.1%	17	2860	*Ochrobactrum*	2.8%	2.8%	1.1%	1.0%	2.5%	3.0%	1	16098
	Others	2.5%	2.0%	1.5%	1.6%	0.7%	0.6%	51	10908	Total	100%	100%	100%	100%	100%	100%	1460	732096
	Total	100%	100%	100%	100%	100%	100%	1460	732096									
Fungi	Ascomycota	74.2%	73.2%	81.8%	81.2%	83.3%	76.2%	505	1183101	Others	41.4%	33.0%	28.9%	27.7%	11.8%	9.4%	351	383122
	Basidiomycota	13.5%	8.7%	7.4%	8.3%	13.5%	19.0%	95	177697	Unclassified	23.9%	20.6%	31.2%	30.3%	8.6%	6.4%	305	304354
	Mortierellomycota	5.7%	8.5%	0.9%	0.9%	1.1%	1.6%	24	47257	*Cladosporium*	4.1%	6.2%	9.6%	8.7%	7.2%	8.7%	4	111807
	Chytridiomycota	0.3%	0.5%	0.1%	0.1%	0.1%	0.1%	7	2795	*Mycosphaerella*	3.9%	13.1%	10.1%	5.0%	34.3%	45.8%	1	282503

Olpidiomycota	0.2%	0.2%	0.1%	0.1%	0.1%	0.1%	2	1831	*Fusarium*	8.8%	5.2%	4.3%	4.1%	1.9%	1.3%	13	64648
Zoopagomycota	0.1%	0.1%	0.0%	0.0%	0.0%	0.0%	1	632	*Alternaria*	2.6%	4.2%	8.3%	6.9%	12.3%	10.1%	5	111727
Mucoromycota	0.0%	0.0%	0.0%	0.0%	0.0%	0.0%	2	344	*Filobasidium*	2.2%	4.6%	2.4%	1.6%	13.1%	8.0%	7	80358
Rozellomycota	0.0%	0.0%	0.0%	0.0%	0.0%	0.0%	1	293	*Aureobasidium*	2.1%	4.3%	0.7%	0.4%	5.2%	5.3%	1	45448
Unclassified	6.0%	8.6%	9.6%	9.3%	1.9%	2.9%	99	96418	*Mortierella*	8.4%	5.5%	0.3%	0.2%	1.6%	1.1%	18	43034
Total	100%	100%	100%	100%	100%	100%	736	1510368	*Penicillium*	1.9%	1.5%	3.7%	3.8%	0.6%	0.4%	27	30048
									Epichloë	0.0%	0.5%	0.0%	10.9%	0.0%	0.0%	1	28921
									Udeniomyces	0.6%	1.3%	0.4%	0.2%	3.7%	3.5%	3	24398
									Total	100%	100%	100%	100%	100%	100%	736	1510368

Supplement excel tables titles:

Table S2. The matrix of bacterial OTUs

Table S3. The matrix of fungal OTUs

Table S4. Detected metabolites of EI and EF seeds

Table S5. Detected metabolites of EI and EF glumes

Table S6. Differential metabolites of EI and EF seeds

Table S7. Differential metabolites of EI and EF glumes

Table S8. Spearman's correlation networks between seeds epiphytic bacterial phyla and seeds metabolites

Table S9. Spearman's correlation networks between seeds epiphytic fungal phyla and seeds metabolites

Table S10. Spearman's correlation networks between seeds endophytic bacterial phyla and seeds metabolites

Table S11. Spearman's correlation networks between seeds endophytic fungal phyla and seeds metabolites

Table S12. Spearman's correlation networks between glumes epiphytic bacterial phyla and glumes metabolites

Table S13. Spearman's correlation networks between glumes epiphytic fungal phyla and glumes metabolites
